# Supplementary material for: Identification of photoperiod-induced specific miRNAs in the adrenal glands of Sunite sheep (Ovis aries)
Source: Front Vet Sci. 2022 Jul 22;9:888207. doi: 10.3389/fvets.2022.888207 (PMC9354845; doi:10.3389/fvets.2022.888207)
Supplement: Supplementary file 1 [file Data_Sheet_1.ZIP › Supplementary file/miRDeep2 file.zip!/miRDeep2 file/Know_Structure/SP42A1/oar-mir-487b.pdf]

miRBase precursor : oar-mir-487b
Total read count : 2774
oar-miR-487b-5p read count : 34
oar-miR-487b-3p read count : 2739
remaining reads : 1

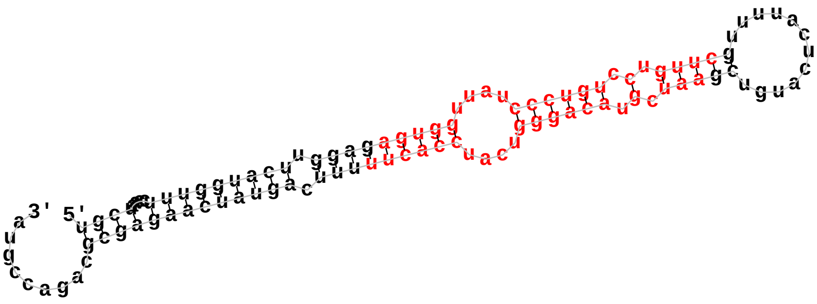

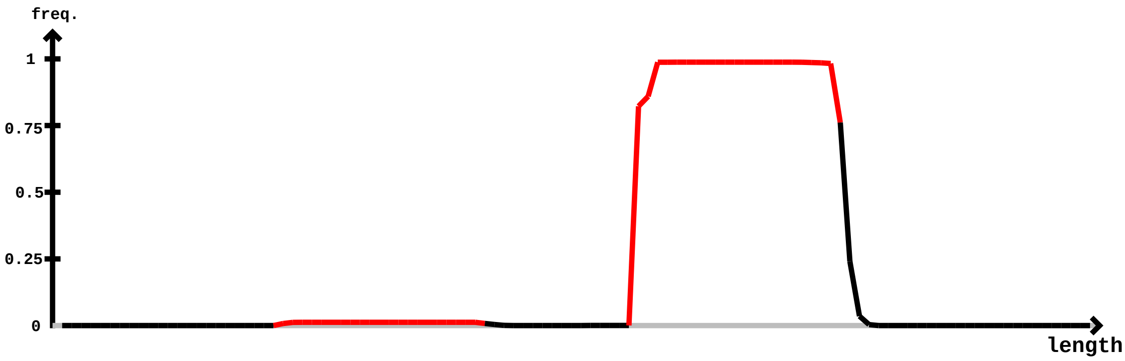


```
                                                                                   oar-miR-487b-3p
                          oar-miR-487b-5p
5'- ugcuaaccuuugguacuuggagagugguuaucccuguccuguucguuuuacucaugucgaaucguacagggucauccacuuuuucaguaucaagagcgcagaccgua  -3'   exp
    .(((....((((((((.((((((((....((((((.(.(((((............))))).).))))))....))))))))).))))))))))).........   reads   mm   sample
    ......................agugguuaucccuguccuguu................................................................   11   0   seq
    ......................agugguuaucccuguccuguuc...............................................................   8   0   seq
    ......................agugguuaucccuguccuguucg..............................................................   1   0   seq
    ......................agugguuaucccuguccuguucgu.............................................................   1   0   seq
    .......................gugguuaucccuguccuguu................................................................   1   0   seq
    .......................gugguuaucccuguccuguuc...............................................................   2   0   seq
    .......................gugguuaucccuguccuguucg..............................................................   8   0   seq
    .......................gugguuaucccuguccuguucgu.............................................................   1   0   seq
    ........................ugguuaucccuguccuguuc...............................................................   1   0   seq
    ......................................................ugucgaaucguacagggucauc...............................   1   0   seq
    ...........................................................aaucguacagggucaucc..............................   2   0   seq
    ...........................................................aaucguacagggucaucca.............................   3   0   seq
    ...........................................................aaucguacagggucauccac............................   6   0   seq
    ...........................................................aaucguacagggucauccacu...........................   598   0   seq
    ...........................................................aaucguacagggucauccacuu..........................   1397   0   seq
    ...........................................................aaucguacagggucauccacuuu.........................   271   0   seq
    ...........................................................aaucguacagggucauccacuuuu........................   3   0   seq
    ............................................................aucguacagggucaucc..............................   1   0   seq
    ............................................................aucguacagggucauccacu...........................   13   0   seq
    ............................................................aucguacagggucauccacuu..........................   31   0   seq
    ............................................................aucguacagggucauccacuuu.........................   48   0   seq
    ............................................................aucguacagggucauccacuuuu........................   10   0   seq
    .............................................................ucguacagggucauccac............................   1   0   seq
    .............................................................ucguacagggucauccacu...........................   3   0   seq
    .............................................................ucguacagggucauccacuu..........................   15   0   seq
    .............................................................ucguacagggucauccacuuu.........................   251   0   seq
    .............................................................ucguacagggucauccacuuuu........................   75   0   seq
    .............................................................ucguacagggucauccacuuuuu.......................   10   0   seq
    ...............................................................guacagggucauccacu...........................   1   0   seq
```
